# Novel polymorphisms in *CYP4A22* associated with susceptibility to coronary heart disease

**DOI:** 10.1186/s12920-024-01833-7

**Published:** 2024-03-04

**Authors:** Kang Huang, Tianyi Ma, Qiang Li, Zanrui Zhong, Yilei Zhou, Wei Zhang, Ting Qin, Shilin Tang, Jianghua Zhong, Shijuan Lu

**Affiliations:** 1 Department of cardiovascular medicine, Central South University Xiangya School of Medicine Affiliated Haikou Hospital, No. 43, Renmin Avenue, Haidian Island, 570100 Haikou, Hainan, China; 2 Medical College, Jingchu University of Technology, Jingmen, Hubei China

**Keywords:** Coronary heart disease, CYP4A22, Genetic polymorphism, Chinese Han population, Susceptibility

## Abstract

**Background:**

Coronary heart disease (CHD) has become a worldwide public health problem. Genetic factors are considered important risk factors for CHD. The aim of this study was to explore the correlation between CYP4A22 gene polymorphism and CHD susceptibility in the Chinese Han population.

**Methods:**

We used SNPStats online software to complete the association analysis among 962 volunteers. False-positive report probability analysis was used to confirm whether a positive result is noteworthy. Haploview software and SNPStats were used for haplotype analysis and linkage disequilibrium. Multi-factor dimensionality reduction was applied to evaluate the interaction between candidate SNPs.

**Results:**

In overall and some stratified analyses (male, age ≤ 60 years or CHD patients complicated with hypertension), *CYP4A22*-rs12564525 (overall, OR = 0.83, *p-value* is 0.042) and *CYP4A22*-rs2056900 (overall, OR = 1.22, *p-value* is 0.032) were associated with the risk of CHD. CYP4A22-4926581 was associated with increased CHD risk only in some stratified analyses. FPRP indicated that all positive results in our study are noteworthy findings. In addition, MDR showed that the single-locus model composed of rs2056900 is the best model for predicting susceptibility to CHD.

**Conclusion:**

There are significant associations between susceptibility to CHD and *CYP4A22* rs12564525, and rs2056900.

**Supplementary Information:**

The online version contains supplementary material available at 10.1186/s12920-024-01833-7.

## Introduction

Coronary heart disease (CHD) refers to coronary atherosclerotic heart disease, also known as ischemic heart disease, which is a narrowing or obstruction of the coronary arteries caused by myocardial ischemia, hypoxia, or necrosis. CHD has become a worldwide public health problem. The total prevalence of CHD in adults in the United States is 6.7%, and the prevalence of CHD is higher in men than in women [1]. The prevalence of CHD in China is also rising [2]. Studies have found that Asians have a higher risk of coronary heart disease and its complications than Western populations due to factors such as genetic factors, higher levels of obesity, or insulin resistance [3]. Genetic factors are considered important risk factors for CHD [2]. With the rapid development of molecular biology and the progress of the human genome project, it has become a hot topic in medical research to find the susceptibility genes of CHD and to elucidate the pathogenesis of CHD from the genetic level.

CYP4A and CYP4F families are isozymes that primarily metabolize arachidonic acid (AA) to 20-hydroxyeicosatetraenoic acid (20-HETE) [4]. 20-HETE can promote the proliferation of vascular endothelial cells [5–7]. In addition, exogenous administration of 20-HETE can inhibit coronary vasodilation caused by vascular endothelial hyperpolarization factors, aggravate the degree of cardiac injury caused by coronary ischemia, and may lead to CHD [8]. Cheng et al. found that overexpression of the CYP4F2 gene could increase the level of 20-HETE, thus promoting the angiogenesis of endothelial cells [9]. Yu et al. confirmed that 20-HETE agonists or overexpression of CYP4A11 could promote angiogenesis, while 20-HETE inhibitors can inhibit the above effects [10]. The above studies have shown that high levels of 20-HETE affect the occurrence and development of CHD, and the level of 20-HETE can be regulated by CYP4A or CYP4F family genes.

Genetic polymorphisms can cause differences in different individuals, and people with specific genetic polymorphisms are more likely to develop related diseases. CYP4 family genes that have been reported to be associated with CHD susceptibility mainly include CYP4A11 [11–13], CYP4F2 [14], etc. CYP4A22 and CYP4A11 are both CYP4A subtypes located on chromosome 1 [15]. To date, the association between CYP4A22 gene polymorphism and CHD susceptibility has not been reported.

The purpose of this study was to investigate the correlation between CYP4A22 gene polymorphism and susceptibility to CHD among the Chinese Han population. It will lay a scientific foundation for further exploring the pathogenesis of CHD in the Chinese Han population, and provide new ideas for finding potential treatments for CHD.

## Materials and methods

### Sample source

A total of 962 volunteers were recruited from Central South University Xiangya School of Medicine Affiliated Haikou Hospital in this study. Among them, 481 patients with CHD were included in the case group. All patients underwent coronary angiography and were diagnosed as CHD by two-experienced interventional cardiologists. CHD was defined by at least one of the three main coronary arteries or their major branches having severe coronary stenosis (≥ 50%) via a coronary angiography [16]. The exclusion criteria of case groups were as follows: (1) patients with liver and kidney transplantation or dysfunction; (2) patients with malignant tumors; (3) patients with thyroid disease; (4) stroke patients; (5) pregnancy or lactation women. The control group was composed of healthy individuals who underwent physical examination in the same hospital. The inclusion criteria of the control group were as follows:: (1) blood routine, urine routine, coagulation function, chest X tube and blood biochemistry, and ECG examination were normal; (2) without liver and kidney diseases; (3) without malignant tumor; (4) non-pregnant or breastfeeding.

After recruiting the two groups of volunteers, we obtained the basic information such as name, age, and gender of all volunteers by consulting medical records and questionnaires. This study was approved by the ethics committee of Central South University Xiangya School of Medicine Affiliated Haikou Hospital. Before blood collection, we fully informed all subjects of the purpose and significance of this study and obtained their informed consent. Then 5 mL of peripheral venous blood was taken.

### Selection of SNPs

We searched the chromosome position of *CYP4A22* in e!Ensembl genome browser (e!GRCh37: http://asia.ensembl.org/Homo_sapiens/Info/Index). We then downloaded the documents related to *CYP4A22* genetic variation among the Beijing population of China in the ‘VCF to PED converter’ function module of the online software. We imported the documents downloaded into Haploview software and set specific parameters in the Tagger module to screen tagSNPs (Tagger R^2^ > 0.8, Min Genotype > 75%, MAF > 0.05, and HWE > 0.01), which helped us narrow the scope of this study. Finally, we randomly selected four genetic polymorphisms from the screened tagSNPs for this study (rs76011927, rs12564525, rs2056900, and rs4926581).

The dbSNP (https://www.ncbi.nlm.nih.gov/snp/), Haploreg (https://pubs.broadinstitute.org/mammals/haploreg/haploreg.php), RegulomeDB (https://regulome.stanford.edu/regulome-search/), Polyphen (http://genetics.bwh.harvard.edu/pph2/) databases were applied to identify the potential functional SNPs in the human *CYP4A22* gene.

### Genotyping

Whole blood DNA was extracted and purified through GoldMag Co. Ltd. DNA reagent kit (Xi’an, China). MassARRAY ® -IPLEX SNP genotyping technology was used for genotyping.

### Data analysis

In this study, the measurement data were expressed as mean ± standard deviation, and count data was expressed as frequency (percentage). The features between CHD patients and healthy controls were compared using χ^2^ and T tests, as appropriate. The χ² goodness-of-fit was used to test whether candidate genetic polymorphisms were consistent with Hardy-Weinberg equilibrium (HWE *P-value* > 0.05 was considered to be consistent with HWE). SNPStats online software (https://www.snpstats.net/start.htm?q=snpstats/start.htm) was applied to assess the association between susceptibility to CHD and candidate genetic polymorphism. Odds ratio (OR) and 95% confidence intervals (CI) values were used to determine the association between genetic polymorphisms and susceptibility to CHD. In order to avoid the interference of confounding factors on the results of association analysis, the results were adjusted for confounding factors (age, sex, drinking, and smoking). Interaction analysis with of *CYP4A22* polymorphisms with covariate (age, gender, smoking and drinking) was performed using SNPStats. Using the SangerBox database to visualize the significant results of the stratified analysis and the forest map was drawn (https://www.snpstats.net/start.htm?q=snpstats/start.htm) [17]. In addition, since multiple hypothesis tests may increase the false positive probability, we used FPRP (false-positive report probability) to detect whether a positive result is noteworthy (FPRP threshold is 0.2 and prior probability level is 0.25). Moreover, we performed LASSO (least absolute shrinkage and selection operator) regression analysis on the data of candidate SNPs and CHD using the R-4.1.1 software package. LASSO is the only attribute for the absolute value of the penalty regression coefficient [18]. The greater the penalty, the greater the contraction of the coefficient, which in turn eliminates unimportant covariates [19, 20]. Taking the threshold lambda (λ) as the geometric mean of the minimum error value and one times the standard deviation, a regression coefficient of significant SNPs can be obtained. Multi-factor dimensionality reduction (MDR) was chosen to assess the effect of SNP-SNP interaction on susceptibility to CHD. SPSS 22.0 software was used for all statistical analyses. A *p-value* less than 0.05 indicates that the results are statistically significant.

## Results

### Basic information of study object

A total of 481 CHD cases (320 males, 161 females, age: 62.38 ± 0.46 years) and 481 healthy individuals (315 males, 166 females, age: 61.22 ± 0.41 years) were included. There was no significant difference in age (*p-value* 0.064), gender (*p-value* 0.785), smoking (*p-value* 0.747) and drinking (*p-value* 0.529) between the two groups, indicating that samples of the two groups were comparable. There were statistically significant differences in the average distribution of red blood cell count (RBC), platelet count (PLT), uric acid (UC), total cholesterol (TC), low-density lipoprotein (LDL), and other clinical indicators (*p* < 0.05). The average levels of RBC, PLT, UC, TC, and LDL in case group were significantly lower than those in control group. There was no significant difference in the distribution of high-density lipoprotein (HDL) (*p-value is* 0.853) (can be seen in Table 1) between the two groups.

**Table 1:** Characteristics of patients with CHD and healthy individuals

Characteristics		Case	Control	***p***
		*n* = 481	*n* = 481	
Age (years)	Mean ± SD	62.38 ± 0.46	61.22 ± 0.41	0.064 ^a^
	> 60	273 (56.8%)	274 (57.0%)	
	≤ 60	208 (43.2%)	207 (43.0%)	
Gender	Male	320 (66.5%)	315 (65.5%)	0.785 ^b^
	Female	161 (33.5%)	166 (34.5%)	
Smoking	Yes	252 (52.4%)	258 (53.6%)	0.747 ^b^
	No	229 (47.6%)	223 (46.4%)	
Drinking	Yes	174 (36.2%)	192 (39.9%)	0.259 ^b^
	No	307 (63.8%)	289 (60.1%)	
CHD complicated with diabetes	Yes	143 (29.7%)	-	-
	No	338 (70.3%)	-	
CHD complicated with hypertension	Yes	299 (62.6%)	-	-
	No	182 (37.8%)	-	
RBC		4.20 ± 0.97	4.81 ± 0.46	< 0.001 ^a^
Hemoglobin		129.47 ± 28.53	147.29 ± 16.50	< 0.001 ^a^
PLT		182.41 ± 75.09	213.36 ± 59.65	< 0.001 ^a^
UC		300.78 ± 91.34	319.95 ± 78.24	< 0.001 ^a^
TC		4.07 ± 1.05	4.76 ± 0.91	< 0.001 ^a^
HDL		1.11 ± 0.26	1.17 ± 0.37	0.853 ^a^
LDL		2.43 ± 0.95	2.61 ± 0.72	< 0.001 ^a^

CHD, coronary heart disease; SD, standard deviation;

RBC, red blood cell count; PLT, platelet count; UC, uric acid; TC, total cholesterol; HDL, high density lipoprotein; LDL, low density lipoprotein

^a^ represents the *p* value calculated by the t-test;

^b^ represents the *p* value calculated by the chi-square test;

‘*p*-value < 0.05’ represents statistical significance

### Genotyping and allele distribution

*CYP4A22* is located on chromosome 1: 47,137,435 − 47,149,727. The chromosome position of *CYP4A22*-rs76011927, -12,564,525, -rs2056900, and -rs4926581 are on chromosome 1: 47,137,516, chromosome 1: 471,414,609, chromosome 1: 47,142,113, and chromosome 1: 47,143,311, respectively. Genotyping of four candidate SNPs had been completed (rs76011927, rs12564525, rs2056900, and rs4926581), and these SNPs met HWE (Table 2: HWE *P* > 5%). The minor allele frequencies (MAF) of candidate SNPs in the African, European, and Han Chinese in the Beijing population (1000 genomes) were determined by e!Ensembl genome browser. The results showed that the MAF of candidate SNPs was different in populations with different genetic backgrounds (Table 2). MAFs of four candidate SNPs were all greater than 5% in study subjects.

**Table 2:** The basic information and HWE about the candidate SNPs of *CYP4A22*.

SNP ID	Function	Chr: Position	Alleles (A/B)	MAF		AF			HWE (***P*** Value)	dbSNP func annot	HaploReg v4.1	RegulomeDB	Polyphen
				Cases	Controls	CHB	AFR	EUR					
rs76011927	missense variant	1: 47,137,516	T/C	0.063	0.064	0.058	-	0.006	0.436	R (Arg) > C (Cys)	Promoter histone marks, Enhancer histone marks, Motifs changed	TF binding + chromatin accessibility peak	BENIGN
rs12564525	missense variant	1: 47,141,609	C/T	0.398	0.444	0.602	0.225	0.206	0.519	R (Arg) > W (Trp)	Motifs changed, Selected eQTL hits	eQTL/caQTL + TF binding / chromatin accessibility peak	BENIGN
rs2056900	missense variant	1: 47,142,113	A/G	0.527	0.478	0.539	0.207	0.193	0.315	G (Gly) > S (Ser)	Motifs changed, Selected eQTL hits	eQTL/caQTL + TF binding / chromatin accessibility peak	BENIGN
rs4926581	missense variant	1: 47,143,311	T/G	0.520	0.480	0.539	0.206	0.193	0.171	V (Val) > I (Ile)	-	eQTL/caQTL + TF binding / chromatin accessibility peak	BENIGN

A: minor allele; B: wild-type allele; SNP: Single nucleotide polymorphisms; MAF: minor allele frequency; AF: allele frequency; CHB: Han Chinese in Beijing, China; EUR: European; AFR: African; HWE: Hardy–Weinberg equilibrium; TF: transcription factor

HWE *P* value > 0.05 indicates that the genotypes were in Hard-weinberg Equilibrium

‘-’: data missing

dbSNP (https://www.ncbi.nlm.nih.gov/snp/), Haploreg (https://pubs.broadinstitute.org/mammals/haploreg/haploreg.php); RegulomeDB (https://regulome.stanford.edu/regulome-search/); Polyphen (http://genetics.bwh.harvard.edu/pph2/)

Database analysis presented that the potential functions of these SNPs might be related to promoter /enhancer histone marks, changed motifs changed, selected expression quantitative trait loci (eQTL) hits, transcription factor (TF) binding, and chromatin accessibility peak. Moreover, all these SNPs rs76011927 R (Arg) > C (Cys), rs12564525 R (Arg) > W (Trp), rs2056900 G (Gly) > S (Ser), and rs4926581 V (Val) > I (Ile) were the BENIGN variants.

### ***CYP4A22*** SNPs associated with susceptibility to CHD in the overall analysis

The overall analysis (Table 3) showed that there are two candidate SNPs associated with susceptibility to CHD. *CYP4A22*-12564525 and -rs2056900 are associated with susceptibility to CHD. Specifically, *CYP4A22*-12564525 had an association with reduced risk of CHD under the allele (OR (95%CI) = OR, 0.83; 95%CI, 0.69–0.99, *p-value is* 0.042), codominant (OR, 0.68; 95%CI, 0.47-1.00; *p-*value is 0.049), and log-additive (OR, 0.68; 95%CI, 0.47-1.00; *p-*value is 0.049) models. *CYP4A22*-rs2056900 was significantly associated with increased risk of CHD (allele: OR, 1.22; 95%CI, 1.02–1.46; *p-*value is 0.032; codominant: OR, 1.49; 95%CI, 1.04–2.14; *p-*value is 0.032; recessive: OR, 1.42; 95%CI, 1.06–1.91, *p*-value is 0.020; and log-additive: OR, 1.22; 95%CI, 1.02–1.46; *p*-value is 0.031).

**Table 3:** Association between candidate SNPs in *CYP4A22* and susceptibility to CHD.

SNP ID	Model	Genotype	Control (*n* = 481)	Case (*n* = 481)	Crude analysis		Adjusted analysis	
					OR (95% CI)	***p***-value ^a^	OR (95% CI)	***p***-value ^b^
rs76011927	Allele	C	900 (93.6%)	901 (93.7%)			1	
		T	62 (6.4%)	61 (6.3%)			0.98 (0.68–1.42)	0.926
	Codominant	CC	422 (87.7%)	422 (87.7%)	1		1	
		CT	56 (11.6%)	57 (11.8%)	1.02 (0.69–1.51)	0.930	1.02 (0.69–1.51)	0.922
		TT	3 (0.6%)	2 (0.4%)	0.67 (0.11–4.01)	0.658	0.60 (0.10–3.64)	0.578
	Dominant	CC	422 (87.7%)	422 (87.7%)	1		1	
		CT-TT	59 (12.3%)	59 (12.3%)	1.00 (0.68–1.47)	0.999	1.00 (0.68–1.47)	0.990
	Recessive	CC-CT	478 (99.4%)	479 (99.6%)	1		1	
		TT	3 (0.6%)	2 (0.4%)	0.67 (0.11-4.00)	0.656	0.60 (0.10–3.62)	0.570
	Overdominant	CC-TT	425 (88.4%)	424 (88.2%)	1		1	
		CT	56 (11.6%)	57 (11.8%)	1.02 (0.69–1.51)	0.920	1.02 (0.69–1.52)	0.910
	Log-additive	-	-	-	0.98 (0.68–1.41)	0.926	0.98 (0.68–1.40)	0.900
rs12564525	Allele	T	535 (55.6%)	579 (60.2%)			1	
		C	427 (44.4%)	383 (39.8%)			0.83 (0.69–0.99)	**0.042**
	Codominant	TT	145 (30.1%)	172 (35.8%)	1		1	
		TC	245 (50.9%)	235 (48.9%)	0.81 (0.61–1.08)	0.143	0.82 (0.61–1.08)	0.160
		CC	91 (18.9%)	74 (15.4%)	0.69 (0.47-1.00)	0.050	0.68 (0.47-1.00)	**0.049**
	Dominant	TT	145 (30.1%)	172 (35.8%)	1		1	
		TC-CC	336 (69.8%)	309 (64.2%)	0.78 (0.59–1.02)	0.064	0.78 (0.59–1.02)	0.070
	Recessive	TT-TC	390 (81.1%)	407 (84.6%)	1		1	
		CC	91 (18.9%)	74 (15.4%)	0.78 (0.56–1.09)	0.147	0.77 (0.55–1.08)	0.130
	Overdominant	TT-CC	236 (49.1%)	246 (51.1%)	1		1	
		TC	245 (50.9%)	235 (48.9%)	0.92 (0.71–1.18)	0.520	0.93 (0.72–1.20)	0.560
	Log-additive	-	-	-	0.82 (0.69–0.99)	**0.040**	0.82 (0.69–0.99)	**0.040**
rs2056900	Allele	G	501 (52.2%)	455 (47.3%)			1	
		A	459 (47.8%)	507 (52.7%)			1.22 (1.02–1.46)	**0.032**
	Codominant	GG	125 (26%)	110 (22.9%)	1		1	
		AG	251 (52.3%)	235 (48.9%)	1.06 (0.78–1.45)	0.697	1.07 (0.78–1.47)	0.661
		AA	104 (21.7%)	136 (28.3%)	1.49 (1.04–2.13)	**0.032**	1.49 (1.04–2.14)	**0.032**
	Dominant	GG	125 (26%)	110 (22.9%)	1		1	
		AG-AA	355 (74%)	371 (77.1%)	1.19 (0.88–1.60)	0.253	1.20 (0.89–1.61)	0.240
	Recessive	GG-AG	376 (78.3%)	345 (71.7%)	1		1	
		AA	104 (21.7%)	136 (28.3%)	1.43 (1.06–1.91)	**0.018**	1.42 (1.06–1.91)	**0.020**
	Overdominant	GG-AA	229 (47.7%)	246 (51.1%)			1	
		AG	251 (52.3%)	235 (48.9%)			0.88 (0.68–1.13)	0.310
	Log-additive	-	-	-	1.22 (1.02–1.46)	**0.031**	1.22 (1.02–1.46)	**0.031**
rs4926581	Allele	G	500 (52.0%)	462 (48.0%)	1		1	
		T	462 (48.0%)	500 (52.0%)	0.87 (0.68–1.12)	0.290	1.17 (0.98–1.40)	0.083
	Codominant	GG	122 (25.4%)	109 (22.7%)	1		1	
		GT	256 (53.2%)	244 (50.7%)	1.07 (0.78–1.46)	0.685	1.08 (0.79–1.47)	0.642
		TT	103 (21.4%)	128 (26.6%)	1.39 (0.96–2.01)	0.077	1.40 (0.97–2.02)	0.076
	Dominant	GG	122 (25.4%)	109 (22.7%)	1		1	
		GT-TT	359 (74.6%)	372 (77.3%)	1.16 (0.86–1.56)	0.327	1.17 (0.87–1.57)	0.300
	Recessive	GG-GT	378 (78.6%)	353 (73.4%)	1		1	
		TT	103 (21.4%)	128 (26.6%)	1.33 (0.99–1.79)	0.060	1.33 (0.98–1.79)	0.063
	Overdominant	GG-TT	225 (46.8%)	237 (49.3%)	1		1	
		GT	256 (53.2%)	244 (50.7%)	0.90 (0.70–1.17)	0.440	0.91 (0.71–1.18)	0.480
	Log-additive	-	-	-	1.18 (0.98–1.42)	0.077	1.18 (0.98–1.42)	0.075

CHD, coronary heart disease; SNP, single nucleotide polymorphisms; OR, odds ratio; CI, confidence interval

“-” indicates log-additive model

^a^*p*-values were calculated by logistic regression analysis

^b^*p*-values were calculated by logistic regression analysis with adjustments for age, gender, smoking, and drinking

‘*p*-value < 0.05’ and bold text represent statistical significance

In the overall analysis, no evidence about the relationship of *CYP4A22*-rs76011927 and -rs4926581 with susceptibility to CHD was found.

### ***CYP4A22*** SNPs associated with susceptibility to CHD in stratified analysis

**Fig. 1:**
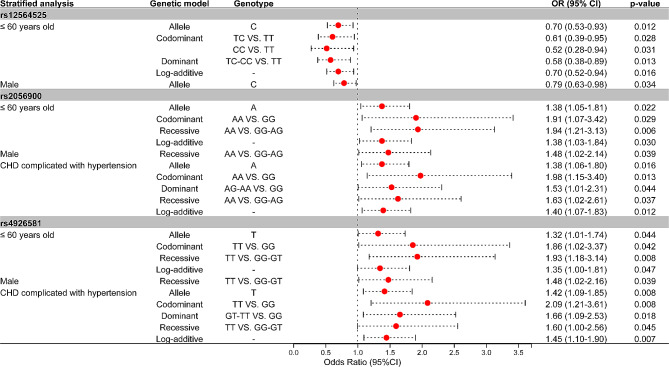
Forest map based on the positive results observed in stratified analysis

As shown in Fig. 1, there is evidence indicating that *CYP4A22*-rs12564525, -rs2056900, and -rs4926581 are associated with susceptibility to CHD in stratified analysis.

#### Stratified analysis of the associations between CYP4A22-rs12564525 and CHD risk

Under the allele (OR, 0.70; *p-value is* 0.012), codominant (OR, 0.61; *p-*value is 0.028 and OR, 0.52; *p-value is* 0.031), dominant (OR, 0.58; *p-value is* 0.013) and log-additive genetic models (OR, 0.70; *p-value is* 0.016), rs12564525 was associated with reduced risk of CHD among participants aged 60 years old or younger. Among male participants, *CYP4A22*-12564525 was a protective genetic factor for CHD (allele: OR, 0.79; *p-value is* 0.034).

#### Stratified analysis of the associations between CYP4A22-rs2056900 and CHD risk

Under the allele (OR, 1.38; *p-value is* 0.022), codominant (OR, 1.91; *p-value is* 0.029), recessive (OR, 1.94; *p-value is* 0.006) and log-additive genetic models (OR, 1.38; *p-value is* 0.030), rs2056900 was related to an increased risk of CHD among participants aged 60 or younger. Among male participants, *CYP4A22*-2056900 was associated with an increased risk of CHD under the recessive genetic model (OR, 1.48; *p-value is* 0.039). In addition, *CYP4A22*-2056900 might be associated with increased risk of CHD complicated with hypertension in the allele (OR, 1.38; *p-value is* 0.016) and codominant (OR, 1.98; *p-value is* 0.013), dominant (OR, 1.53; *p-value is* 0.044), recessive (OR, 1.63; *p-value is* 0.037), and log-additive models (OR, 1.40; *p-value is* 0.012).

#### Stratified analysis of the associations between CYP4A22-rs4926581and CHD risk

Among participants aged 60 years old or younger, -rs4926581 was associated with an increased risk of CHD under the allele (OR, 1.32; *p-value is* 0.044), codominant (OR, 1.86; *p-value is* 0.042), recessive (OR, 1.93; *p-value is* 0.008), and log-additive models (OR, 1.35; *p-value is* 0.047). Among male participants, rs4926581 was related to an increased risk of CHD under the recessive genetic model (OR, 1.48; *p-value is* 0.039). *CYP4A22*-rs4926581 was associated with increased risk of CHD complicated with hypertension under the allele (OR, 1.42; *p-value is* 0.008), codominant (OR, 2.09; *p-value is* 0.008), dominant (OR, 1.66; *p-value is* 0.018), recessive (OR, 1.60; *p-value is* 0.045), and log-additive genetic (OR, 1.45; *p-value is* 0.007) models.

#### Others

There was no association between candidate SNPs and susceptibility to CHD among participants older than 60 years old or female (Supplemental Table 1). In addition to the above, we also grouped participants according to smoking and drinking status (Supplemental Table 2) or stratified the CHD case group according to the presence or absence of diabetes (Supplemental Table 3), but no significant association was found.

#### Interaction analysis with of CYP4A22 polymorphisms with covariate (age, gender, smoking and drinking)

Table 4 displayed the interaction of *CYP4A22* polymorphisms with covariate (age, gender, smoking and drinking). The results displayed interaction analysis of rs76011927 with covariate drinking in the codominant (*p* = 0.021), dominant (*p* = 0.045), overdominant (*p* = 0.019) models. Moreover, the interaction of rs4926581 with covariate gender in the overdominant model (*p* = 0.046) and age in the recessive model (*p* = 0.047) was found.

**Table 4:** Interaction analysis with of *CYP4A22* polymorphisms with covariate (age, gender, smoking and drinking)

SNP ID	Interaction analysis with covariate age				Interaction analysis with covariate gender			
	Codominant	Dominant	Recessive	Overdominant	Codominant	Dominant	Recessive	Overdominant
rs76011927	0.420	0.410	0.270	0.470	0.210	0.250	0.130	0.380
rs12564525	0.160	0.060	0.480	0.230	0.410	0.210	0.930	0.230
rs2056900	0.200	0.075	0.750	0.240	0.370	0.540	0.310	0.170
rs4926581	0.120	0.910	**0.047**	0.140	0.130	0.250	0.230	**0.046**
SNP ID	Interaction analysis with covariate smoking				Interaction analysis with covariate drinking			
	Codominant	Dominant	Recessive	Overdominant	Codominant	Dominant	Recessive	Overdominant
rs76011927	0.800	0.650	0.690	0.580	**0.021**	**0.045**	0.120	**0.019**
rs12564525	0.860	0.620	0.750	0.730	0.780	0.600	0.540	0.980
rs2056900	0.770	0.470	0.910	0.670	0.940	0.730	0.870	0.880
rs4926581	0.900	0.880	0.650	0.850	0.670	0.790	0.370	0.580

SNP, Single nucleotide polymorphism

*p* < 0.05 respects the data is statistically significant

### LASSO regression

LASSO analysis showed that when the threshold λ was 0.024, *CYP4A22*-2056900 was an important covariate for predicting the CHD risk model. Logistic LASSO regression results showed that *CYP4A22*-2056900 (regression coefficient = 0.060) was positively associated with CHD risk.

### FPRP analysis for the positive results

The statistical power for positive results in the overall analysis ranges from 94.1 to 100.0%, indicating that the sample size of this study was large enough to effectively prevent the occurrence of false positive results. FPRP analysis (Supplemental Table 4) showed that all positive results found in our study had a prior probability of less than 0.2 (the prior probability level is 0.25 and the FPRP threshold is 0.2), indicating that the significant associations between candidate SNPs and susceptibility to CHD found in our study are all noteworthy findings.

### Association between clinical indicators level and candidate SNPs

The results (Table 4) displayed that the hemoglobin levels in subjects with homozygous genotype ‘TT’ and heterozygous genotype ‘CT’ of *CYP4A22*-76011927 were lower than those of wild genotype ‘CC’ (*p-value is* 0.022). The red blood cell count in subjects with homozygous genotype ‘CC’ and heterozygous genotype ‘TC’ of *CYP4A22*-12564525 was significantly lower than those of wild genotype ‘TT’ (*p-value is* 0.022). There was no association between the levels of clinical indicators and other candidate SNPs (Supplemental Table 5).

### SNP-SNP interaction and CHD risk

**Fig. 2:**
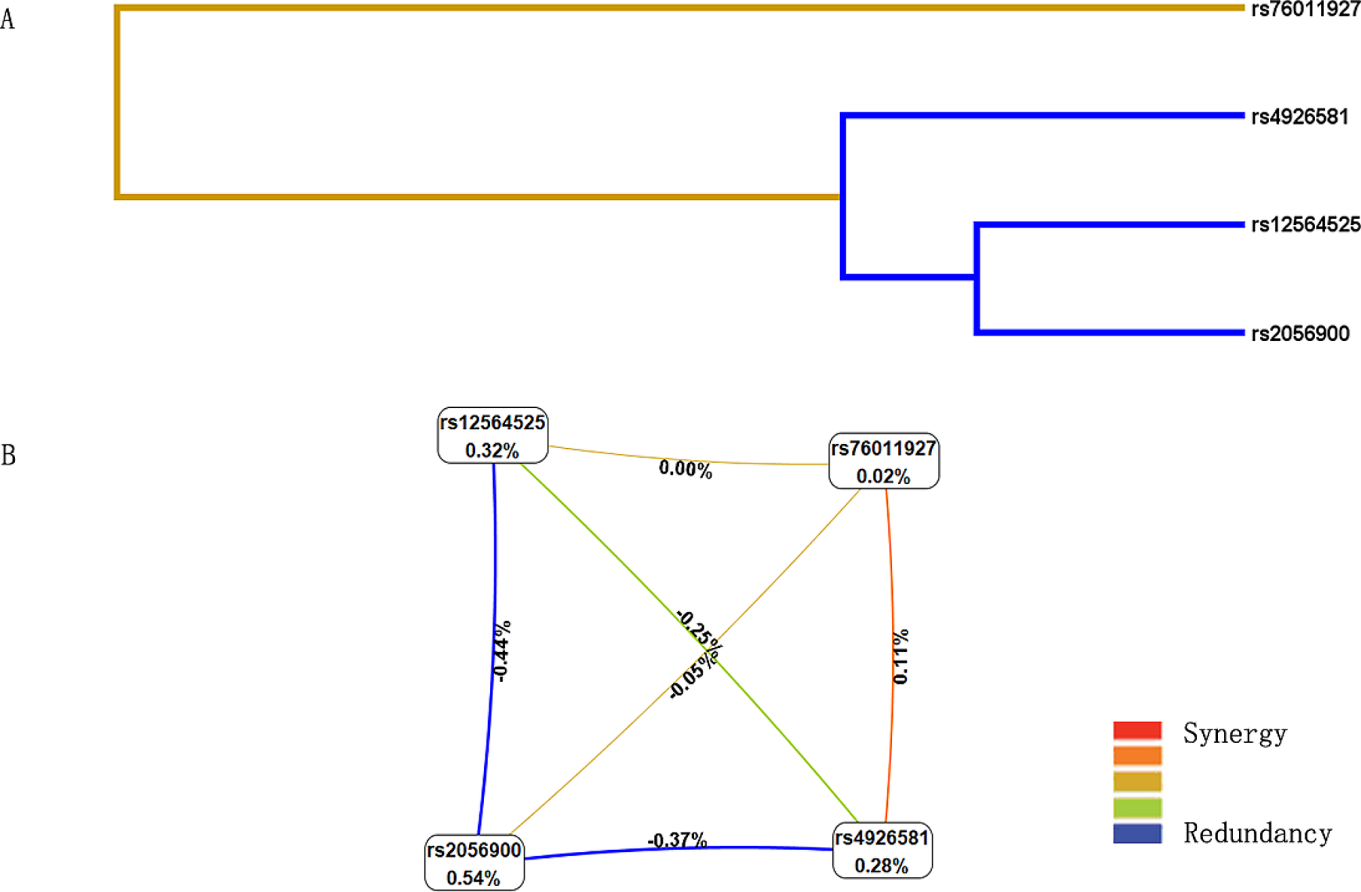
Multifactor dimensionality reduction (MDR) analysis of interaction between the candidate SNPs of *CYP4A22* (rs76011927, rs12564525, rs2056900, and rs4926581). **(A)** SNP-SNP Interaction Dendrogram: the color represents the degree of redundancy or synergy between SNP-SNP; the closer the color is to red, the more synergy, and the closer to blue, the more redundancy. **(B)** Fruchterman-Reingold: values in nodes represent the IGs of individual attribute (main effects). Values between nodes are IGs of each pair of attributes (interaction effects)

MDR analysis(Table 5) showed that the single-locus model composed of rs2056900 could be chosen as the best model for predicting CHD risk (CVC = 9/10 and the highest test accuracy = 0.526). Figure 2A is the dendrogram, showing the interaction between candidate SNPs. In addition, information gain (IG) was used to evaluate the attribute interactions, and the results showed that rs2056900 had the highest IG value.

**Table 5:** Clinical indicators of patients based on the genotypes of selected SNPs

Indicators	rs76011927					rs12564525			
	TT	CT	CC	***p***		TT	TC	CC	***p***
**RBC**	2.8 ± 1.62	4.22 ± 0.86	4.2 ± 0.98	0.123		4.35 ± 0.8	4.14 ± 1.04	4.02 ± 1.07	**0.022**
**Hemoglobin**	74 ± 26.87	129.49 ± 26.91	129.74 ± 28.55	**0.022**		133.38 ± 24.81	127.91 ± 29.89	125.45 ± 31.32	0.068
**PLT**	133 ± 188.09	198.75 ± 81.87	180.43 ± 73.49	0.145		184.37 ± 69.64	180.88 ± 76.57	182.74 ± 83.08	0.899
**UC**	278.5 ± 33.23	307.07 ± 101.23	300.04 ± 90.22	0.814		302.08 ± 82.23	301.43 ± 95.45	295.64 ± 99.26	0.872
**TC**	4.85 ± 1.36	4.01 ± 1	4.07 ± 1.06	0.527		4.12 ± 1.15	4.07 ± 0.99	3.94 ± 1.01	0.503
**HDL**	1.08 ± 0.18	1.09 ± 0.24	1.12 ± 0.27	0.829		1.13 ± 0.27	1.12 ± 0.26	1.08 ± 0.27	0.402
**LDL**	2.72 ± 0.16	2.58 ± 0.91	2.41 ± 0.95	0.444		2.44 ± 0.88	2.42 ± 1	2.41 ± 0.92	0.965

RBC, red blood cell count; PLT, platelet count; UC, uric acid; TC, total cholesterol; HDL, high density lipoprotein; LDL, low density lipoprotein

‘*p*-value < 0.05’ and bold text represent statistical significance

**Table 6:** *CYP4A22* SNP–SNP interaction models analyzed by the MDR method

Model	Training Bal. Acc	Testing Bal. Acc	OR (95% CI)	p-value	CVC
rs2056900	0.533	0.526	1.42 (1.13–1.78)	**0.0027**	9/10
rs76011927, rs2056900	0.539	0.507	1.44 (1.14–1.80)	**0.0018**	7/10
rs76011927, rs12564525, rs2056900	0.543	0.510	1.48 (1.18–1.86)	**0.0007**	6/10
rs76011927, rs12564525, rs2056900, rs4926581	0.546	0.513	1.61 (1.28–2.02)	**< 0.0001**	10/10

MDR, multifactor dimensionality reduction; Bal. Acc., balanced accuracy; CVC, cross-validation consistency; OR, odds ratio; 95% CI, 95% confidence interval

*p* values were calculated using χ^2^ tests; ‘*p-*value < 0.05’ and bold text represent statistical significance

## Discussion

In this study, we investigated the association between CYP4A22 genetic polymorphism and CHD susceptibility in 962 participants. This study found evidence that three missense variants in CYP4A22 (rs12564525 and rs2056900) were associated with CHD susceptibility. In particular, CYP4A22-rs12564525, and-rs2056900 were associated with CHD susceptibility in the overall analysis and stratified analysis. This study is the first to analyze the association between CYP4A22 genetic polymorphism and CHD susceptibility in the Chinese Han population, and FPRP results suggest that all the positive results found in this study are noteworthy new findings.

Coronary artery disease is known to be an important cause of death, but it is preventable [21]. In recent years, genome-wide association studies have successfully identified many genetic factors affecting the risk of CHD, but the discovered genetic polymorphisms still cannot fully explain the heritability of common CHD [22]. Therefore, the identification of CHD susceptibility loci in specific populations will be helpful for clinical individualized treatment of CHD., The results showed thatrs12564525 and rs2056900 were protective and risk genetic factors in overall subjects, males, or subjects aged less than or equal to 60 years. More importantly, the results of MDR and LASSO analysis further support that CYP4A22-rs2056900 is closely related to CHD susceptibility. However, previous studies have shown that increasing age is a risk factor for CHD [23], and it is confirmed that men are more likely to develop CHD than women [21]. Based on the above research reports and the results of this study, we speculate that CYP4A22-rs12564525 and CYP4A22-rs2056900 are associated with CHD susceptibility in the study subjects and the effect of environmental risk factors need to be further assessed. In addition, we also found evidence that CYP4A22-rs4926581 was associated with increased CHD risk. Although the above correlation was only found in the stratified analysis, the FPRP results showed that the above positive results were noteworthy new findings. In any case, further verification tests are necessary, which will make the results of this study more convincing. By referring to relevant literature, we only found reports on the molecular function and clinical significance of CYP4 family gene polymorphisms [24] in one study, but no reports on the association of CYP4A22-rs12564525 and -rs2056900 with disease susceptibility were found. This study is the first to find evidence that CYP4A22-rs12564525, -rs2056900, -rs4926581 are associated with CHD susceptibility in the Chinese Han population.

In both patients with chronic or acute coronary syndrome, 20-HETE levels were significantly higher than those in the control group [25, 26]. These results suggest that 20-HETE may play an important role in CHD and myocardial ischemic injury. 20-HETE can promote angiogenesis through endothelial progenitor cells (EPCs). Cardiac angiogenesis is not only a physiological response to ischemia or hypoxia but also a potential target for therapeutic strategies [27]. Studies have shown that CYP4A22 gene is expressed in EPCs and can generate 20-HETE [28]. 20-HETE agonists or overexpression of CYP4A11 can promote angiogenesis, while 20-HETE inhibitors can inhibit the effects [10]. Combined with previous studies and the results of this study, we speculate that the the mutant allele ‘A' of CYP4A22-rs12564525 may reduce the expression level of CYP4A22, thereby reducing the level of 20-HETE, and further reducing the risk of CHD in the study subjects. Similarly, CYP4A22-rs2056900 and-rs4926581 may also increase the level of 20-HETE by regulating the expression level of CYP4A22, thus further increasing the risk of CHD in the study subjects. However, the above is only speculation, and further functional verification experiments are necessary. In any case, this study provides a reliable theoretical basis for the mechanism of CYP4A22 in the development of CHD. At the same time, it provides new ideas for risk assessment, clinical individualized prevention and treatment of CHD in the Chinese Han population.

There are some limitations in this study. In order to ensure the reliability and repeatability of the results, it is necessary to conduct large sample sizes or validation studies in populations with different genetic backgrounds. Secondly, the role of CYP4A22 genetic polymorphism in the occurrence and development of CHD requires further functional verification experiments to further verify the effects of CYP4A22-rs12564525, -rs2056900, -rs4926581 on the levels of CYP4A22 and 20-HETE in CHD patients. This study contributes to the development of 20-HETE as a potential treatment for CHD. Thirdly, the correlation between these tagSNPs and CHD prognosis need to further explore in the future. Fourth, the population sizes of the different groups for the stratification analyses were limited. So, larger sample size are needed to verify our finding. In any case, this study is the first to explore the correlation between CYP4A22 genetic polymorphism and CHD susceptibility in the Chinese Han population.

## Conclusion

In summary, the results of this study suggested that CYP4A22-rs12564525, -rs2056900, -rs4926581 were associated with coronary heart disease susceptibility. In particular, there is sufficient evidence that CYP4A22-rs2056900 is associated with an increased risk of CHD in the Chinese Han population.

### Electronic supplementary material

Below is the link to the electronic supplementary material.

Supplementary Material 1

## Data Availability

The datasets generated and/or analyzed during the current study are available in the [Zenodo] repository, 10.5281/zenodo.7543554.
