# Supplementary material for: Novel polymorphisms in CYP4A22 associated with susceptibility to coronary heart disease
Source: BMC Med Genomics. 2024 Mar 4;17:66. doi: 10.1186/s12920-024-01833-7 (PMC10913669; doi:10.1186/s12920-024-01833-7)
Supplement: Supplementary file 1 — Supplementary Material 1 [file 12920_2024_1833_MOESM1_ESM.docx]

**Supplemental table 1** Association between candidate SNPs in *CYP4A22* and susceptibility to CHD in stratified analysis (Age and geneder).

| **SNP ID** | **Model** | **Genotype** | **> 60 years old**  **(case: 273 vs control: 274)** | | **≤ 60 years old**  **(case: 208 vs control: 207)** | |  | **Female**  **(case: 161 vs control: 166)** | | **Males**  **(case: 320 vs control: 315)** | |
| --- | --- | --- | --- | --- | --- | --- | --- | --- | --- | --- | --- |
|  |  |  | **OR (95% CI)** | **p-value** | **OR (95% CI)** | **p-value** |  | **OR (95% CI)** | **p-value** | **OR (95% CI)** | **p-value** |
| rs76011927 | Allele | C | 1 |  | 1 |  |  | 1 |  | 1 |  |
|  |  | T | 1.13 (0.70-1.83) | 0.614 | 0.81 (0.46-1.43) | 0.459 |  | 1.39 (0.75-2.57) | 0.298 | 0.81 (0.52-1.29) | 0.376 |
|  | Codominant | CC | 1 |  | 1 |  |  | 1 |  | 1 |  |
|  |  | CT | 1.15 (0.67-1.95) | 0.614 | 0.89 (0.48-1.62) | 0.694 |  | 1.23 (0.62-2.44) | 0.554 | 0.92 (0.56-1.52) | 0.748 |
|  |  | TT | 0.91 (0.12-6.78) | 0.928 | NA | - |  | NA | - | 0.30 (0.03-2.93) | 0.298 |
|  | Dominant | CC | 1 |  | 1 |  |  | 1 |  | 1 |  |
|  |  | CT-TT | 1.13 (0.68-1.89) | 0.640 | 0.85 (0.47-1.56) | 0.610 |  | 1.29 (0.65-2.54) | 0.460 | 0.87 (0.54-1.42) | 0.580 |
|  | Recessive | CC-CT | 1 |  | 1 |  |  | 1 |  | 1 |  |
|  |  | TT | 0.90 (0.12-6.67) | 0.920 | NA | - |  | NA | 0.240 | 0.30 (0.03-2.95) | 0.270 |
|  | Overdominant | CC-TT | 1 |  | 1 |  |  | 1 |  | 1 |  |
|  |  | CT | 1.15 (0.68-1.95) | 0.610 | 0.89 (0.49-1.63) | 0.710 |  | 1.22 (0.61-2.42) | 0.570 | 0.93 (0.56-1.53) | 0.770 |
|  | Log-additive | - | 1.10 (0.69-1.77) | 0.690 | 0.83 (0.46-1.48) | 0.520 |  | 1.34 (0.69-2.58) | 0.380 | 0.84 (0.53-1.32) | 0.450 |
| rs12564525 | Allele | T | 1 |  | 1 |  |  | 1 |  | 1 |  |
|  |  | C | 0.94 (0.74-1.2) | 0.616 | 0.70 (0.53-0.93) | **0.012** |  | 0.92 (0.67-1.25) | 0.588 | 0.79 (0.63-0.98) | **0.034** |
|  | Codominant | TT | 1 |  | 1 |  |  | 1 |  | 1 |  |
|  |  | TC | 0.97 (0.66-1.42) | 0.881 | 0.61 (0.39-0.95) | **0.028** |  | 1.37 (0.81-2.30) | 0.242 | 0.73 (0.51-1.04) | 0.077 |
|  |  | CC | 0.84 (0.51-1.39) | 0.500 | 0.52 (0.28-0.94) | **0.031** |  | 0.89 (0.44-1.78) | 0.739 | 0.67 (0.42-1.08) | 0.098 |
|  | Dominant | TT | 1 |  | 1 |  |  | 1 |  | 1 |  |
|  |  | TC-CC | 0.93 (0.65-1.34) | 0.710 | 0.58 (0.38-0.89) | **0.013** |  | 1.22 (0.75-2.00) | 0.420 | 0.71 (0.51-1.00) | **0.048** |
|  | Recessive | TT-TC | 1 |  | 1 |  |  | 1 |  | 1 |  |
|  |  | CC | 0.86 (0.55-1.34) | 0.500 | 0.71 (0.42-1.20) | 0.200 |  | 0.74 (0.40-1.37) | 0.340 | 0.82 (0.54-1.24) | 0.340 |
|  | Overdominant | TT-CC | 1 |  | 1 |  |  | 1 |  | 1 |  |
|  |  | TC | 1.03 (0.73-1.45) | 0.870 | 0.77 (0.52-1.13) | 0.180 |  | 1.42 (0.89-2.26) | 0.140 | 0.83 (0.61-1.14) | 0.260 |
|  | Log-additive | - | 0.93 (0.73-1.18) | 0.540 | 0.70 (0.52-0.94) | **0.016** |  | 1.00 (0.72-1.40) | 0.980 | 0.80 (0.64-1.01) | 0.062 |
| rs2056900 | Allele | G | 1 |  | 1 |  |  | 1 |  | 1 |  |
|  |  | A | 1.11 (0.87-1.4) | 0.397 | 1.38 (1.05-1.81) | **0.022** |  | 1.26 (0.93-1.72) | 0.136 | 1.19 (0.96-1.49) | 0.116 |
|  | Codominant | GG | 1 |  | 1 |  |  | 1 |  | 1 |  |
|  |  | AG | 1.13 (0.74-1.72) | 0.573 | 0.98 (0.60-1.58) | 0.922 |  | 1.60 (0.91-2.83) | 0.104 | 0.90 (0.61-1.32) | 0.583 |
|  |  | AA | 1.25 (0.78-2.01) | 0.363 | 1.91 (1.07-3.42) | **0.029** |  | 1.38 (0.73-2.62) | 0.327 | 1.37 (0.87-2.17) | 0.177 |
|  | Dominant | GG | 1 |  | 1 |  |  | 1 |  | 1 |  |
|  |  | AG-AA | 1.17 (0.79-1.73) | 0.440 | 1.19 (0.75-1.88) | 0.460 |  | 1.52 (0.89-2.58) | 0.120 | 1.02 (0.71-1.48) | 0.900 |
|  | Recessive | GG-AG | 1 |  | 1 |  |  | 1 |  | 1 |  |
|  |  | AA | 1.15 (0.78-1.69) | 0.470 | 1.94 (1.21-3.13) | **0.006** |  | 1.02 (0.60-1.72) | 0.950 | 1.48 (1.02-2.14) | **0.039** |
|  | Overdominant | GG-AA | 1 |  | 1 |  |  | 1 |  | 1 |  |
|  |  | AG | 1.01 (0.72-1.42) | 0.970 | 0.71 (0.48-1.05) | 0.088 |  | 1.36 (0.86-2.17) | 0.190 | 0.77 (0.56-1.05) | 0.097 |
|  | Log-additive | - | 1.12 (0.88-1.42) | 0.360 | 1.38 (1.03-1.84) | **0.030** |  | 1.17 (0.85-1.62) | 0.330 | 1.17 (0.93-1.47) | 0.180 |
| rs4926581 | Allele | G | 1 |  | 1 |  |  | 1 |  | 1 |  |
|  |  | T | 1.07 (0.84-1.35) | 0.586 | 1.32 (1.01-1.74) | **0.044** |  | 1.17 (0.86-1.59) | 0.309 | 1.17 (0.94-1.46) | 0.161 |
|  | Codominant | GG | 1 |  | 1 |  |  | 1 |  | 1 |  |
|  |  | GT | 1.17 (0.77-1.79) | 0.454 | 0.95 (0.58-1.54) | 0.826 |  | 1.76 (1.00-3.12) | 0.051 | 0.87 (0.59-1.28) | 0.478 |
|  |  | TT | 1.16 (0.72-1.88) | 0.542 | 1.85 (1.02-3.37) | **0.042** |  | 1.19 (0.62-2.29) | 0.602 | 1.34 (0.85-2.14) | 0.210 |
|  | Dominant | GG | 1 |  | 1 |  |  | 1 |  | 1 |  |
|  |  | GT-TT | 1.17 (0.79-1.74) | 0.440 | 1.14 (0.71-1.80) | 0.590 |  | 1.54 (0.90-2.63) | 0.110 | 0.99 (0.68-1.44) | 0.960 |
|  | Recessive | GG-GT | 1 |  | 1 |  |  | 1 |  | 1 |  |
|  |  | TT | 1.04 (0.71-1.54) | 0.830 | 1.93 (1.18-3.14) | **0.008** |  | 0.82 (0.48-1.40) | 0.470 | 1.48 (1.02-2.16) | **0.039** |
|  | Overdominant | GG-TT | 1 |  | 1 |  |  | 1 |  | 1 |  |
|  |  | GT | 1.09 (0.77-1.53) | 0.630 | 0.71 (0.48-1.05) | 0.083 |  | 1.62 (1.01-2.59) | 0.054 | 0.75 (0.55-1.03) | 0.074 |
|  | Log-additive | - | 1.08 (0.85-1.37) | 0.550 | 1.35 (1.00-1.81) | **0.047** |  | 1.09 (0.79-1.51) | 0.590 | 1.16 (0.92-1.46) | 0.210 |

CHD, coronary heart disease; SNP, single nucleotide polymorphisms; OR, odds ratio; CI, confidence interval; NA, not applicable.

“-” indicates log-additive model.

*p*-values were calculated by logistic regression analysis with adjustments for age, gender, smoking, and drinking.

‘*p*-value < 0.05’ and bold text represent statistical significance.

**Supplemental table 2** Association between candidate SNPs in *CYP4A22* and susceptibility to CHD in stratified analysis (Smoking and drinking).

| **SNP ID** | **Model** | **Genotype** | **Smoking**  **(case: 252vs control: 258)** | | **Non-smoking**  **(case: 229 vs control: 223)** | |  | **Drinking**  **(case: 174 vs control: 192)** | | **Non-drinking**  **(case: 307 vs control: 289)** | |
| --- | --- | --- | --- | --- | --- | --- | --- | --- | --- | --- | --- |
|  |  |  | **OR (95% CI)** | ***p*-value** | **OR (95% CI)** | ***p*-value** |  | **OR (95% CI)** | ***p*-value** | **OR (95% CI)** | ***p*-value** |
| rs76011927 | Allele | C | 1 |  | 1 |  |  | 1 |  | 1 |  |
|  |  | T | 0.91 (0.53-1.57) | 0.744 | 1.04 (0.63-1.7) | 0.890 |  | 0.65 (0.35-1.22) | 0.176 | 1.24 (0.78-1.96) | 0.370 |
|  | Codominant | CC | 1 |  | 1 |  |  | 1 |  | 1 |  |
|  |  | CT | 0.86 (0.48-1.55) | 0.610 | 1.12 (0.65-1.94) | 0.682 |  | 0.56 (0.28-1.09) | 0.088 | 1.49 (0.89-2.49) | 0.133 |
|  |  | TT | 0.85 (0.05-13.81) | 0.908 | 0.47 (0.04-5.44) | 0.548 |  | N/A | - | 0.26 (0.03-2.53) | 0.244 |
|  | Dominant | CC | 1 |  | 1 |  |  | 1 |  | 1 |  |
|  |  | CT-TT | 0.86 (0.48-1.53) | 0.600 | 1.08 (0.63-1.84) | 0.780 |  | 0.59 (0.31-1.15) | 0.120 | 1.35 (0.82-2.23) | 0.230 |
|  | Recessive | CC-CT | 1 |  | 1 |  |  | 1 |  | 1 |  |
|  |  | TT | 0.86 (0.05-14.05) | 0.920 | 0.47 (0.04-5.35) | 0.530 |  | NA | - | 0.24 (0.02-2.42) | 0.190 |
|  | Overdominant | CC-TT | 1 |  | 1 |  |  | 1 |  | 1 |  |
|  |  | CT | 0.86 (0.48-1.55) | 0.610 | 1.13 (0.65-1.95) | 0.670 |  | 0.55 (0.28-1.08) | 0.078 | 1.50 (0.90-2.51) | 0.120 |
|  | Log-additive | - | 0.87 (0.50-1.50) | 0.610 | 1.03 (0.63-1.70) | 0.900 |  | 0.65 (0.34-1.22) | 0.180 | 1.21 (0.76-1.92) | 0.420 |
| rs12564525 |  | T | 1 |  | 1 |  |  | 1 |  | 1 |  |
|  |  | C | 0.80 (0.62-1.02) | 0.074 | 0.86 (0.66-1.12) | 0.277 |  | 0.77 (0.57-1.03) | 0.076 | 0.87 (0.69-1.1) | 0.238 |
|  | Codominant | TT | 1 |  | 1 |  |  | 1 |  | 1 |  |
|  |  | TC | 0.79 (0.53-1.17) | 0.237 | 0.88 (0.57-1.36) | 0.566 |  | 0.75 (0.47-1.2) | 0.229 | 0.85 (0.59-1.22) | 0.368 |
|  |  | CC | 0.61 (0.35-1.06) | 0.077 | 0.77 (0.45-1.32) | 0.344 |  | 0.58 (0.31-1.1) | 0.094 | 0.73 (0.45-1.19) | 0.208 |
|  | Dominant | TT | 1 |  | 1 |  |  | 1 |  | 1 |  |
|  |  | TC-CC | 0.74 (0.51-1.08) | 0.120 | 0.85 (0.57-1.27) | 0.420 |  | 0.70 (0.45-1.10) | 0.120 | 0.82 (0.58-1.15) | 0.250 |
|  | Recessive | TT-TC | 1 |  | 1 |  |  | 1 |  | 1 |  |
|  |  | CC | 0.70 (0.43-1.15) | 0.160 | 0.83 (0.52-1.34) | 0.440 |  | 0.70 (0.40-1.22) | 0.200 | 0.81 (0.52-1.25) | 0.340 |
|  | Overdominant | TT-CC | 1 |  | 1 |  |  | 1 |  | 1 |  |
|  |  | TC | 0.92 (0.65-1.31) | 0.650 | 0.97 (0.67-1.42) | 0.890 |  | 0.90 (0.60-1.37) | 0.630 | 0.94 (0.68-1.30) | 0.710 |
|  | Log-additive | - | 0.78 (0.60-1.02) | 0.067 | 0.88 (0.67-1.15) | 0.340 |  | 0.76 (0.56-1.03) | 0.080 | 0.85 (0.67-1.08) | 0.190 |
| rs2056900 |  | G | 1 |  | 1 |  |  | 1 |  | 1 |  |
|  |  | A | 1.16 (0.91-1.49) | 0.229 | 1.28 (0.99-1.67) | 0.061 |  | 1.26 (0.94-1.69) | 0.119 | 1.19 (0.95-1.49) | 0.134 |
|  | Codominant | GG | 1 |  | 1 |  |  | 1 |  | 1 |  |
|  |  | AG | 1.10 (0.70-1.73) | 0.666 | 1.05 (0.67-1.65) | 0.829 |  | 1.06 (0.64-1.78) | 0.817 | 1.09 (0.73-1.63) | 0.669 |
|  |  | AA | 1.35 (0.81-2.26) | 0.255 | 1.61 (0.95-2.74) | 0.079 |  | 1.61 (0.89-2.93) | 0.118 | 1.48 (0.93-2.36) | 0.099 |
|  | Dominant | GG | 1 |  | 1 |  |  | 1 |  | 1 |  |
|  |  | AG-AA | 1.18 (0.77-1.81) | 0.450 | 1.21 (0.79-1.85) | 0.380 |  | 1.22 (0.75-1.98) | 0.430 | 1.21 (0.83-1.77) | 0.330 |
|  | Recessive | GG-AG | 1 |  | 1 |  |  | 1 |  | 1 |  |
|  |  | AA | 1.26 (0.84-1.89) | 0.270 | 1.56 (1.00-2.44) | 0.051 |  | 1.55 (0.95-2.52) | 0.078 | 1.39 (0.96-2.03) | 0.084 |
|  | Overdominant | GG-AA | 1 |  | 1 |  |  | 1 |  | 1 |  |
|  |  | AG | 0.94 (0.66-1.34) | 0.730 | 0.84 (0.58-1.23) | 0.370 |  | 0.84 (0.55-1.27) | 0.400 | 0.90 (0.65-1.24) | 0.500 |
|  | Log-additive | - | 1.16 (0.90-1.51) | 0.250 | 1.26 (0.97-1.64) | 0.085 |  | 1.27 (0.94-1.71) | 0.120 | 1.22 (0.96-1.54) | 0.097 |
| rs4926581 |  | G | 1 |  | 1 |  |  | 1 |  | 1 |  |
|  |  | T | 1.14 (0.89-1.45) | 0.312 | 1.22 (0.94-1.58) | 0.140 |  | 1.27 (0.95-1.7) | 0.104 | 1.11 (0.89-1.4) | 0.354 |
|  | Codominant | GG | 1 |  | 1 |  |  | 1 |  | 1 |  |
|  |  | GT | 1.08 (0.69-1.69) | 0.730 | 1.08 (0.69-1.71) | 0.730 |  | 1.04 (0.62-1.74) | 0.884 | 1.1 (0.74-1.65) | 0.639 |
|  |  | TT | 1.29 (0.77-2.17) | 0.330 | 1.49 (0.87-2.57) | 0.148 |  | 1.67 (0.91-3.04) | 0.096 | 1.3 (0.81-2.09) | 0.277 |
|  | Dominant | GG | 1 |  | 1 |  |  | 1 |  | 1 |  |
|  |  | GT-TT | 1.14 (0.75-1.75) | 0.530 | 1.20 (0.78-1.84) | 0.410 |  | 1.21 (0.74-1.97) | 0.440 | 1.16 (0.79-1.70) | 0.450 |
|  | Recessive | GG-GT | 1 |  | 1 |  |  | 1 |  | 1 |  |
|  |  | TT | 1.22 (0.81-1.85) | 0.330 | 1.42 (0.90-2.23) | 0.130 |  | 1.62 (0.99-2.65) | 0.052 | 1.22 (0.83-1.78) | 0.320 |
|  | Overdominant | GG-TT | 1 |  | 1 |  |  | 1 |  | 1 |  |
|  |  | GT | 0.94 (0.66-1.35) | 0.750 | 0.90 (0.62-1.31) | 0.590 |  | 0.81 (0.53-1.23) | 0.310 | 0.97 (0.70-1.34) | 0.830 |
|  | Log-additive | - | 1.14 (0.88-1.48) | 0.320 | 1.22 (0.93-1.59) | 0.160 |  | 1.29 (0.96-1.74) | 0.095 | 1.14 (0.90-1.44) | 0.280 |

CHD, coronary heart disease; SNP, single nucleotide polymorphisms; OR, odds ratio; CI, confidence interval; NA, not applicable.

“-” indicates log-additive model.

*p*-values were calculated by logistic regression analysis with adjustments for age, gender, smoking, and drinking.

‘*p*-value < 0.05’ and bold text represent statistical significance.

**Supplemental table 3** Association between candidate SNPs in *CYP4A22* and susceptibility to CHD complicated with diabetes.

| **SNPs** | **Model** | **Genotype** | **CHD complicated with diabetes** | | | |
| --- | --- | --- | --- | --- | --- | --- |
|  |  |  | **No (n=338)** | **Yes (n=143)** | **OR (95% CI)** | ***p*-value** |
| rs76011927 | Allele | C | 636 (94.1%) | 265 (92.7%) | 1 |  |
|  |  | T | 40 (5.9%) | 21 (7.3%) | 1.26 (0.73-2.18) | 0.407 |
|  | Codominant | CC | 300 (88.8%) | 122 (85.3%) | 1 |  |
|  |  | CT | 36 (10.7%) | 21 (14.7%) | 1.38 (0.77-2.48) | 0.279 |
|  |  | TT | 2 (0.6%) | 0 (0%) | NA | - |
|  | Dominant | CC | 300 (88.8%) | 122 (85.3%) | 1 |  |
|  |  | CT-TT | 38 (11.2%) | 21 (14.7%) | 1.31 (0.73-2.34) | 0.370 |
|  | Recessive | CC-CT | 336 (99.4%) | 143 (100%) | 1 |  |
|  |  | TT | 2 (0.6%) | 0 (0%) | NA | - |
|  | Overdominant | CC-TT | 302 (89.3%) | 122 (85.3%) | 1 |  |
|  |  | CT | 36 (10.7%) | 21 (14.7%) | 1.39 (0.78-2.49) | 0.270 |
|  | Log-additive | - | --- | --- | 1.22 (0.70-2.12) | 0.490 |
| rs12564525 | Allele | T | 413 (61.1%) | 166 (58.0%) | 1 |  |
|  |  | C | 263 (38.9%) | 120 (42.0%) | 1.14 (0.86-1.5) | 0.377 |
|  | Codominant | TT | 123 (36.4%) | 49 (34.3%) | 1 |  |
|  |  | TC | 167 (49.4%) | 68 (47.5%) | 1.03 (0.66-1.59) | 0.913 |
|  |  | CC | 48 (14.2%) | 26 (18.2%) | 1.33 (0.74-2.39) | 0.337 |
|  | Dominant | TT | 123 (36.4%) | 49 (34.3%) | 1 |  |
|  |  | TC-CC | 215 (63.6%) | 94 (65.7%) | 1.09 (0.72-1.65) | 0.670 |
|  | Recessive | TT-TC | 290 (85.8%) | 117 (81.8%) | 1 |  |
|  |  | CC | 48 (14.2%) | 26 (18.2%) | 1.31 (0.77-2.23) | 0.320 |
|  | Overdominant | TT-CC | 171 (50.6%) | 75 (52.5%) | 1 |  |
|  |  | TC | 167 (49.4%) | 68 (47.5%) | 0.94 (0.63-1.39) | 0.750 |
|  | Log-additive | - | --- | --- | 1.13 (0.85-1.50) | 0.410 |
| rs2056900 | Allele | G | 366 (54.1%) | 141 (49.3%) | 1 |  |
|  |  | A | 310 (45.9%) | 145 (50.7%) | 1.21 (0.92-1.6) | 0.169 |
|  | Codominant | GG | 98 (29%) | 38 (26.6%) | 1 |  |
|  |  | AG | 170 (50.3%) | 65 (45.5%) | 0.99 (0.62-1.59) | 0.975 |
|  |  | AA | 70 (20.7%) | 40 (28%) | 1.47 (0.85-2.52) | 0.168 |
|  | Dominant | GG | 98 (29%) | 38 (26.6%) | 1 |  |
|  |  | AG-AA | 240 (71%) | 105 (73.4%) | 1.13 (0.73-1.76) | 0.580 |
|  | Recessive | GG-AG | 268 (79.3%) | 103 (72%) | 1 |  |
|  |  | AA | 70 (20.7%) | 40 (28%) | 1.47 (0.94-2.32) | 0.098 |
|  | Overdominant | GG-AA | 168 (49.7%) | 78 (54.5%) | 1 |  |
|  |  | AG | 170 (50.3%) | 65 (45.5%) | 0.83 (0.56-1.24) | 0.360 |
|  | Log-additive | - | --- | --- | 1.21 (0.92-1.59) | 0.180 |
| rs4926581 | Allele | G | 362 (53.6%) | 138 (48.3%) | 1 |  |
|  |  | T | 314 (46.4%) | 148 (51.7%) | 1.24 (0.94-1.63) | 0.133 |
|  | Codominant | GG | 94 (27.8%) | 34 (23.8%) | 1 |  |
|  |  | GT | 174 (51.5%) | 70 (49%) | 1.11 (0.68-1.8) | 0.677 |
|  |  | TT | 70 (20.7%) | 39 (27.3%) | 1.53 (0.87-2.66) | 0.138 |
|  | Dominant | GG | 94 (27.8%) | 34 (23.8%) | 1 |  |
|  |  | GT-TT | 244 (72.2%) | 109 (76.2%) | 1.23 (0.78-1.94) | 0.370 |
|  | Recessive | GG-GT | 268 (79.3%) | 104 (72.7%) | 1 |  |
|  |  | TT | 70 (20.7%) | 39 (27.3%) | 1.42 (0.90-2.25) | 0.130 |
|  | Overdominant | GG-TT | 164 (48.5%) | 73 (51%) | 1 |  |
|  |  | GT | 174 (51.5%) | 70 (49%) | 0.91 (0.61-1.34) | 0.620 |
|  | Log-additive | - | --- | --- | 1.23 (0.93-1.64) | 0.140 |

CHD, coronary heart disease; SNP, single nucleotide polymorphisms; OR, odds ratio; CI, confidence interval; NA, not applicable.

“-” indicates log-additive model.*p*-values were calculated by logistic regression analysis with adjustments for age, gender, smoking, and drinking.

*p*-value < 0.05’ and bold text represent statistical significance.

**Supplemental table 4** The FPRP and statistical power analysis for all the positive results.

| **SNP ID** | **Model** | **Genotype** | **OR (95%CI)** | ***p*** | **S****tatistical Power ^a^ (%)** | **Prior probability** | | |
| --- | --- | --- | --- | --- | --- | --- | --- | --- |
|  |  |  |  |  |  | **0.25** | **0.1** | **0.01** |
| **Overall analysis** |  |  |  |  |  |  |  |  |
| rs12564525 | Allele | C | 0.83 (0.69-0.99) | 0.042 | 100.0 | 0.103 ^b^ | 0.256 | 0.791 |
|  | Codominant | CC | 0.68 (0.47-1.00) | 0.049 | 94.1 | 0.137 ^b^ | 0.324 | 0.840 |
|  | Log-additive | - | 0.82 (0.69-0.99) | 0.040 | 100.0 | 0.105 ^b^ | 0.260 | 0.794 |
| rs2056900 | Allele | A | 1.22 (1.02-1.46) | 0.032 | 100.0 | 0.083 ^b^ | 0.213 | 0.748 |
|  | Codominant | AA | 1.49 (1.04-2.14) | 0.032 | 94.4 | 0.089 ^b^ | 0.227 | 0.764 |
|  | Recessive | AA | 1.42 (1.06-1.91) | 0.020 | 98.8 | 0.058 ^b^ | 0.157 ^b^ | 0.672 |
|  | Log-additive | - | 1.22 (1.02-1.46) | 0.031 | 100.0 | 0.083 ^b^ | 0.213 | 0.748 |
| **≤ 60 years old** |  |  |  |  |  |  |  |  |
| rs12564525 | Allele | C | 0.70 (0.53-0.93) | 0.012 | 99.0 | 0.040 ^b^ | 0.112 ^b^ | 0.581 |
|  | Codominant | TC | 0.61 (0.39-0.95) | 0.028 | 81.1 | 0.096 ^b^ | 0.242 | 0.778 |
|  |  | CC | 0.52 (0.28-0.94) | 0.031 | 55.2 | 0.142 ^b^ | 0.332 | 0.845 |
|  | Dominant | TC-CC | 0.58 (0.38-0.89) | 0.013 | 75.2 | 0.048 ^b^ | 0.132 ^b^ | 0.625 |
|  | Log-additive | - | 0.70 (0.52-0.94) | 0.016 | 98.7 | 0.051 ^b^ | 0.139 ^b^ | 0.640 |
| rs2056900 | Allele | A | 1.38 (1.05-1.81) | 0.022 | 99.6 | 0.057 ^b^ | 0.153 ^b^ | 0.665 |
|  | Codominant | AA | 1.91 (1.07-3.42) | 0.029 | 56.2 | 0.136 ^b^ | 0.321 | 0.839 |
|  | Recessive | AA | 1.94 (1.21-3.13) | 0.006 | 55.0 | 0.035 ^b^ | 0.098 ^b^ | 0.544 |
|  | Log-additive | - | 1.38 (1.03-1.84) | 0.030 | 99.4 | 0.078 ^b^ | 0.203 | 0.737 |
| rs4926581 | Allele | T | 1.32 (1.01-1.74) | 0.044 | 99.8 | 0.128 ^b^ | 0.306 | 0.829 |
|  | Codominant | TT | 1.86 (1.02-3.37) | 0.042 | 59.5 | 0.170 ^b^ | 0.381 | 0.871 |
|  | Recessive | TT | 1.93 (1.18-3.14) | 0.008 | 55.7 | 0.042 ^b^ | 0.116 ^b^ | 0.590 |
|  | Log-additive | - | 1.35 (1.00-1.81) | 0.047 | 99.6 | 0.119 ^b^ | 0.288 | 0.817 |
| **Male** |  |  |  |  |  |  |  |  |
| rs12564525 | Allele | C | 0.79 (0.63-0.98) | 0.034 | 100.0 | 0.088 ^b^ | 0.224 | 0.760 |
| rs2056900 | Recessive | AA | 1.48 (1.02-2.14) | 0.039 | 94.5 | 0.106 ^b^ | 0.261 | 0.796 |
| rs4926581 | Recessive | TT | 1.48 (1.02-2.16) | 0.039 | 94.1 | 0.118 ^b^ | 0.287 | 0.816 |
| **CHD complicated with hypertension** |  |  |  |  |  |  |  |  |
| rs2056900 | Allele | A | 1.38 (1.06-1.80) | 0.016 | 99.7 | 0.050 ^b^ | 0.136 ^b^ | 0.635 |
|  | Codominant | AA | 1.98 (1.15-3.40) | 0.013 | 51.5 | 0.072 ^b^ | 0.188 ^b^ | 0.719 |
|  | Dominant | AG-AA | 1.53 (1.01-2.31) | 0.044 | 89.9 | 0.126 ^b^ | 0.301 | 0.826 |
|  | Recessive | AA | 1.63 (1.02-2.61) | 0.037 | 80.3 | 0.135 ^b^ | 0.320 | 0.838 |
|  | Log-additive | - | 1.40 (1.07-1.83) | 0.012 | 99.5 | 0.040 ^b^ | 0.111 ^b^ | 0.579 |
| rs4926581 | Allele | T | 1.42 (1.09-1.85) | 0.008 | 99.4 | 0.027 ^b^ | 0.078 ^b^ | 0.483 |
|  | Codominant | TT | 2.09 (1.21-3.61) | 0.008 | 43.7 | 0.053 ^b^ | 0.144 ^b^ | 0.650 |
|  | Dominant | GT-TT | 1.66 (1.09-2.53) | 0.018 | 80.7 | 0.064 ^b^ | 0.170 ^b^ | 0.693 |
|  | Recessive | TT | 1.60 (1.00-2.56) | 0.045 | 82.4 | 0.154 ^b^ | 0.353 | 0.857 |
|  | Log-additive | - | 1.45 (1.10-1.90) | 0.007 | 99.0 | 0.021 ^b^ | 0.060 ^b^ | 0.414 |

FPRP: false-positive report probability.

^a^ Statistical power ^a^ was calculated using the number of observations in the subgroup and the OR and p values in this table.

^b^ The level of false-positive report probability threshold was set at 0.2, ‘^b^’ indicate noteworthy findings.

**Supplemental table 5** Clinical indicators of patients based on the genotypes of selected SNPs.

| **Indicators** | **rs2056900** | | | |  | **rs4926581** | | | |
| --- | --- | --- | --- | --- | --- | --- | --- | --- | --- |
|  | AA | AG | GG | p |  | TT | GT | GG | p |
| **RBC** | 4.3 ± 0.82 | 4.22 ± 0.99 | 4.03 ± 1.09 | 0.089 |  | 4.3 ± 0.07 | 4.22 ± 0.06 | 4.03 ± 0.11 | 0.089 |
| **Hemoglobin** | 132.03 ± 25.56 | 129.88 ± 28.22 | 125.47 ± 32.25 | 0.195 |  | 131.94 ± 2.32 | 130 ± 1.79 | 125.41 ± 3.1 | 0.200 |
| **PLT** | 182.72 ± 69.45 | 181.76 ± 73.15 | 183.44 ± 85.89 | 0.980 |  | 183.84 ± 6.25 | 181.34 ± 4.65 | 183.15 ± 8.25 | 0.949 |
| **UC** | 298.14 ± 81.03 | 304.03 ± 93.6 | 297.08 ± 98.95 | 0.749 |  | 300.87 ± 7.12 | 302.43 ± 6.05 | 296.93 ± 9.61 | 0.876 |
| **TC** | 4.02 ± 1.12 | 4.09 ± 0.99 | 4.07 ± 1.11 | 0.825 |  | 3.97 ± 0.1 | 4.12 ± 0.07 | 4.07 ± 0.11 | 0.477 |
| **HDL** | 1.13 ± 0.29 | 1.11 ± 0.25 | 1.1 ± 0.27 | 0.770 |  | 1.13 ± 0.03 | 1.11 ± 0.02 | 1.1 ± 0.03 | 0.757 |
| **LDL** | 2.4 ± 0.92 | 2.39 ± 0.81 | 2.55 ± 1.21 | 0.367 |  | 2.35 ± 0.08 | 2.42 ± 0.05 | 2.54 ± 0.12 | 0.330 |

RBC, red blood cell count; PLT, platelet count; UC, uric acid; TC, total cholesterol; HDL, high density lipoprotein; LDL, low density lipoprotein

*p*-value < 0.05’ and bold text represent statistical significance
